# Assessing ethical competence and ethical climate: development and psychometric testing of the Clinical Ethics Support Measurement Instrument (CES-MI)

**DOI:** 10.1186/s12910-026-01573-6

**Published:** 2026-09-08

**Authors:** Cecilia Bartholdson, Anna Bennesved, Marika Wenemark, Catarina Fischer-Grönlund, Anders Bremer, Margareta Brännström, Pernilla Pergert, Mia Svantesson

**Affiliations:** 1 Department of Women’s and Children’s Health, Karolinska Institutet, Stockholm, Sweden; 2 Astrid Lindgren Children’s Hospital, Region Stockholm, Stockholm Sweden; 3 Faculty of Health and Life Sciences, Linnaeus University, Växjö, Sweden; 4 Department of Ambulance Service, Region Kronoberg, Växjö, Sweden; 5 Centre of Interprofessional Collaboration within Emergency Care, Linnaeus University, Växjö, Sweden; 6 Department of Medicine and Health Sciences, Linköping University, Linköping, Sweden; 7 Department of Nursing, Umeå University, Umeå, Sweden; 8 Centre for Research Ethics & Bioethics, Department of Public Health and Caring Sciences, Uppsala University, Uppsala, Sweden; 9 University Health Care Research Center, Faculty of Medicine and Health, Örebro University, Örebro, Sweden

**Keywords:** Clinical ethics support, Ethical climate, Ethical competence, Ethical challenges, Instrument development, Moral case deliberation, Psychometric testing

## Abstract

**Background:**

Clinical ethics support (CES) is increasingly recognised as essential in healthcare, yet its evaluation remains challenging due to the complexity of CES practices and the latent, psychosocial nature of relevant outcomes. The European Moral Case Deliberation Outcomes Instruments (Euro-MCD original and 2.0) represents important steps in operationalising CES outcomes. However, there remains a need for a further theoretically grounded and psychometrically robust instrument. This study aimed to revise the Euro-MCD 2.0 and to develop and psychometrically test a new measurement instrument for CES.

**Methods:**

A sequential mixed-methods instrument redevelopment and validation design was used, guided by an abductive, theory-driven approach. Content validity processes involved conceptual framing, item revision, item development, and cognitive evaluation. Construct validity processes involved psychometric testing using survey data, incorporating principal component analysis and complementary item-level assessments.

**Results:**

The revision of Euro-MCD 2.0 resulted in the Clinical Ethics Support Measurement Instrument (CES-MI), assessing ethical competence and ethical climate. Ethical competence comprises holding moral judgement, being responsive, and acting with moral courage (9 items, using a 5-point scale assessing perceived difficulty). Ethical climate comprises permissive dialogue, supportive relationships, and managing ethical concerns together (7 items, using a 4-point scale assessing perceived extent). Measurement properties were generally strong: targeting was appropriate, response categories functioned as intended, and only minor issues, such as limited local dependencies were observed. Reliability indices were high, unidimensionality was supported across the constructs, and no differential item functioning was detected by sex or intervention groups.

**Conclusions:**

The CES-MI is a coherent, theoretically grounded, and psychometrically robust instrument for measurement, suitable for both clinical practice and research. The strong validity likely reflects the extensive qualitative and psychometric work underpinning the CES-MI, together with a user-friendly response format that supports reflective and authentic self-assessment. Next steps include examining the instrument’s sensitivity to change over time for example by exploring whether open-ended follow-up questions could deepen understanding of concrete changes, as well as, assessing applicability across diverse healthcare settings.

**Supplementary Information:**

The online version contains supplementary material available at https://doi.org/10.1186/s12910-026-01573-6.

## Background

Healthcare organisations require clinical ethics support (CES) to address the ethical challenges that routinely arise in patient care. CES encompasses a wide range of support, primarily focused on patient cases, including clinical ethics committees, individual ethics consultations, and moral case deliberation [1]. In Sweden, the latter, facilitated case-based group reflections on ethically challenging patient situations, is a common form [2]. However, CES methods vary considerably in their goals, organisational forms, and accessibility, reflecting the absence of a national strategy to harmonise CES. CES has been conceptualised as a complex intervention involving multiple interacting components, context-dependent mechanisms, and diverse stakeholders [3, 4].

Demonstrating the impact of CES is challenging, not only because of the complex psychosocial nature of ethics, but also because it relies on latent construct that are inherently difficult to measure [5–9]. Although numerous qualitative studies report positive user experiences [10, 11], these methods are better suited to generate an in-depth understanding of experiences than to measure the effects of CES. Evaluating CES is essential for ensuring its quality, and this field has gained growing attention in recent years [12–15].

Previous work has laid important groundwork for identifying relevant CES outcomes [16–20]. The European Moral Case Deliberation Outcome Instrument (Euro-MCD) [16] represents a substantial step forward in this regard. Euro-MCD measures perceived importance of potential outcomes before CES and experienced outcomes. A three-country field study [21–23] identified the need to reduce items and domains as well as revise the measurement and response scale, for instance due to ceiling effects, with participants frequently rating outcomes as highly important.

The revised instrument, Euro-MCD 2.0 [17] measures moral competence, moral teamwork and moral action. In Euro-MCD 2.0 moral competence refers to an individual’s moral sensitivity, analytical skills and virtuous attitude. Moral teamwork refers to the collective communication and relational process of an open dialogue and supportive relationships. Moral action includes joint actions of moral decision-making and responsible care [17].

Although Euro-MCD 2.0 represents methodological progress, persistent challenges remain, especially regarding the theoretical grounding. In particular, the concept of moral teamwork generated substantial discussion within the Euro-MCD research team regarding whether it constituted an established concept [17]. Ethical climate was considered as an alternative because of its similarities to findings in the ethics literature, including a study of managers’ experiences of CES [24]. However, ethical climate was considered too broad, and moral teamwork was adopted as a pragmatic choice [17]. The decision to keep moral action separate in Euro-MCD 2.0 also warrants further scrutiny. In the above-mentioned field study of managers’ experiences [24] morally grounded action emerged as one aspect of ethical climate, consistent with Victor and Cullen’s [25] definition. Likewise, moral competence is commonly understood to include action-oriented aspects. Given the breadth of moral teamwork and moral competence, there is a need for concepts that are theoretically robust and more closely aligned with the practice of CES.

Finally, the assessment of inherently norm-laden constructs presents methodological challenges, as responses may be influenced by social desirability and other forms of response distortion [5–9]. Current instruments rarely address the linguistic and cognitive mechanisms through which such bias arises, and the reliability of traditional countermeasures, such as social desirability scales, has been increasingly questioned [7, 26, 27]. This methodological gap, i.e., the absence of valid tools that capture these mechanisms while mitigating bias, remains a significant challenge. Addressing this requires the development of new measures that not only draw on prior conceptual work in CES evaluation but also incorporate strategies that minimise evaluative pressure and enable more authentic self-assessment and responses [27, 28].

In summary, although the Euro-MCD project has made substantial contributions to CES evaluation, there remains a need for theoretically grounded and psychometrically robust instruments capable of capturing the impact of CES. The aim of the present study was therefore to revise the Euro-MCD 2.0 and to develop and psychometrically test a new measurement instrument for CES.

## Methods

### Design

**Fig. 1:**
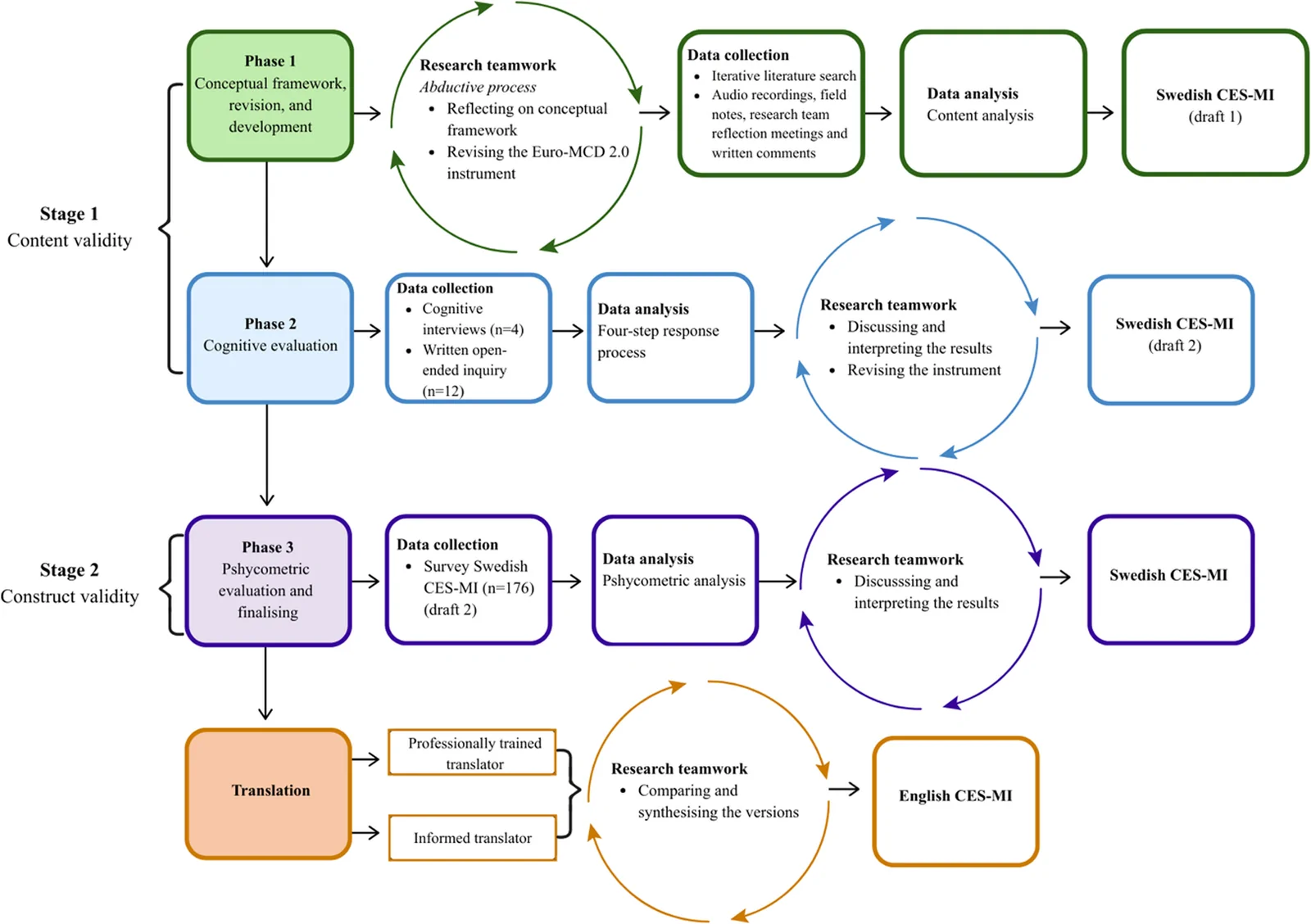
Illustration of stages and phases for development and psychometric evaluation of CES-MI

This study employed a mixed‑methods instrument redevelopment and validation design with three phases of qualitative and quantitative analyses, integrated within an abductive, theory-driven process [29]. The study was conducted through theoretical reasoning and performed in two stages. The first stage (content validity) included: (1) Conceptual framework, revision and development; and (2) Cognitive evaluation. The second stage (Construct validity) included (3) Psychometric evaluation and finalising. The stages were evaluated in a continuous, interactive and joint reflections in a national CES research team (Fig. 1). The new CES measurement instrument is grounded in the Euro-MCD 2.0 [17], permission to use and revise the instrument was requested from, and subsequently granted by, the originators.

### Stage 1: content validity

#### Phase 1 – conceptual framework, revision, and development

##### Research teamwork and data collection

The national CES research team was established, comprising seven registered nurses who were also researchers in the field of CES from five universities with clinical expertise in geriatrics, anaesthesia, intensive care, paediatrics, palliation, and pre-hospital emergency care. The development of the CES-MI was undertaken through systematic joint reflection on Euro-MCD 2.0 domains, subdomains and items. This work was also informed by findings from previous Euro-MCD field studies and by empirical literature and theoretical literature on CES practice [17, 30–35]. Revision and further development of the conceptual framework, building on the Euro-MCD 2.0 domains moral competence, moral teamwork and moral action, followed an abductive and reflective analytic process [36]. In Euro-MCD 2.0 moral competence refers to an individual’s integrated ability to recognise a moral issue, reason through it, and demonstrate the willingness to act on that reasoning while remaining accountable for the outcome. Moral teamwork refers to the collective, relational process of ethical reflection, focusing on the quality of interaction characterised by honest dialogue, equal communication, and social cohesion. Moral action refers to the practical realisation of ethical reflection and is characterised by decisions based on moral grounds and the provision of responsible care that is responsive to patient values [17]. The abductive and reflective analytic process ensured theoretical coherence and alignment with contemporary understandings of CES outcomes. This process supported the delineation of constructs, ethical competence and ethical climate in CES-MI, that reflect individual and organisational dimensions and are grounded in distinct theoretical traditions. Data from the revision process during 2019–2025 consisted of documentation from digital meetings, field notes, and audio‑recordings generated during the 23 reflection meetings. Most meetings lasted approximately three hours, with some ranging from 1.5 to 2 h, supplemented by two full-day in-person meetings. Between meetings, research team members submitted written comments on proposed items revisions, which informed subsequent reflections.

##### Data analysis

Data were analysed using semantic content analysis [37], facilitated by the NVivo software.

#### Phase 2 – cognitive evaluation

##### Participants and data collection

*Cognitive interviews* were conducted to explore how participants comprehended the items, retrieved relevant information, made estimations/evaluations and provided answers [38, 39]. A convenience sampling strategy was employed, with participants recruited through the researchers’ professional networks. Four healthcare professionals participated in the interviews; three men and one woman, including a registered nurse, a physician, and two nurse assistants (providing direct basic care), all with experience of facing ethical challenges in different care contexts. The median age was 61 years (range 53–67), and the median length of work experience was 27 years (range 10–44). Interviews were conducted by three of the authors (AB, MS, CFG) either at the participants’ workplaces or digitally. Participants were instructed to *think aloud* as they answered the first draft of the instrument and retrospective probes were used to clarify participants’ interpretations of concepts and their ability to understand and answer the items. At the end of each interview, participants provided feedback on the overall clarity and relevance of the instrument. All interviews were audio‑recorded and lasted between 25 and 56 min (mean 36 min). *A* written *open-ended inquiry* was also used to examine whether participants’ understanding of ‘ethically difficult situations’ aligned with the intended meaning of this central concept in the new instrument. This step aimed to ensure that the concept would be understood and function as intended for future respondents. Using a convenience sampling strategy, 12 ambulance clinicians were recruited through two of the researchers’ professional networks. The inquiry asked: “Please describe an ethically difficult situation”, and participants were encouraged to provide one or more examples. 

##### Data analysis

The *cognitive interviews* were analysed using the four-step response process model [39], which outlines the cognitive stages respondents engage in when answering items: (1) comprehension of the item, (2) retrieval of relevant information from memory, (3) judgment based on that information, and (4) formulation of a response. Each transcript was examined to understand how participants interpreted the items, accessed and selected relevant experiences, evaluated their answers, and articulated their final responses. The emerging interpretations and potential issues were then discussed within the research team to ensure a shared understanding and to refine the instrument accordingly.

Responses from the *open-ended inquiry* were reviewed and consolidated by identifying overlapping or redundant descriptions [40]. Through this process, the material was reduced to a final set of 28 unique ethically difficult situation.

### Stage 2: construct validity

#### Phase 3 – psychometric evaluation

##### Participants and data collection

All clinically active ambulance clinicians (*n* = 1159) in five Swedish regions were invited to participate via a professional research agency. Because the ambulance departments could not distinguish clinically active clinicians from the full population, email addresses for all staff registered within the departments, including individuals on parental leave, sick leave, or working in administrative roles, were provided to the research agency by the heads of each department. The invitation therefore explicitly stated that participation was intended for clinically active clinicians. Those who accepted the invitation registered through a provided link and submitted their email address. They subsequently received a link to the digital version of CES-MI (draft 2). The research agency administered all reminders and managed the participant follow-up process. Two weeks after the initial distribution, a reminder was sent to those who had not yet registered their email addresses. Two additional reminders were then sent to registered participants who had not completed the questionnaire, at two and three weeks after the survey launch. In total, 183 individuals registered their email addresses. Of these, 176 (96%) completed the questionnaire (Table 1), resulting in a low dropout rate between registration and questionnaire completion. Data collection took place within the context of a larger research project, from which this study draws its sample for the psychometric evaluation of the CES-MI.

**Table 1:** Demographics of the participants (*n* = 176)

Characteristics	
*Sex (n)*	
Male/female	89/87
*Education (n)*	
RN/EMT*	28/12
RN with specialist education**	136
*Age (years)*	
Median	43
Mean	43
Min-Max (range)	24-67
*Work experience as ambulance clinicians (years)*	
Median	10
Mean	12
Min-Max (range)	0,5–41

* RN = Registered Nurse (3 years of education, bachelor’s level), EMT = Emergency Medical Technician (this category includes both Assistant Nurses (general upper secondary education) and Emergency Medical Technicians (additional 1-year specialization in ambulance care). ** Prehospital Emergency Care, Public Health Care, Emergency Care, Intensive Care, Anaesthesia Care (all are 1 year of specialist training, master’s level)

##### Data analysis

Data analysis was undertaken to evaluate the psychometric properties of the two constructs included in the final version of the CES-MI. The unidimensional Rasch measurement model for ordered categories (the unrestricted polytomous Rasch model) was applied [41]. All Rasch analyses were conducted using RUMM2030, version 5.8.1, which employs a conditional maximum likelihood estimation procedure. To assess unidimensionality, FACTOR version 12.4.5 was used. The analysis focused on global and individual item fit, local independence, response category functioning, the person-item threshold distribution, reliability, and differential item function (DIF) for sex and data collection method (intervention group) [41–46]. The significance level for the Rasch analyses was set at *p* < 0.05. Bonferroni corrections were applied when evaluating individual item fit (ethical competence: *p* < 0.006 and ethical climate *p* < 0.007). Internal consistency of the scales was evaluated using Cronbach’s alpha and ordinal alpha. Analyses were performed in R version 4.5.3 (The R Foundation for Statistical Computing, Vienna, Austria) using the psych and haven package. Given the ordinal nature of the Likert-scale data, ordinal alpha based on polychoric correlation matrices was calculated alongside the standard Cronbach’s alpha to provide a more robust estimation of scale reliability [47].

##### Translation

The final Swedish version of the CES-MI was translated into English by one uninformed professional translator and one of the authors (MS), a native Swedish speaker fluent in English. The research team, also fluent in English, then reviewed and discussed alternative English phrasings to ensure that the translated constructs, aspects of constructs and items accurately reflected the intended meaning of the original Swedish version. Following this process, the research team agreed on the instrument’s final English name: the Clinical Ethics Support Measurement Instrument (CES-MI).

## Results

The revision of the Euro-MCD 2.0 instrument led to the development of the CES-MI (Table 2), which measures ethical competence and ethical climate. The results follow the same structure as the method section, with the three distinctive phases (Fig. 1). The CES-MI is available in Supplementary file 1.

### Phase 1 – conceptual framework, revision and development

A key decision was to replace the Euro-MCD 2.0 domain moral teamwork with ethical climate, as highlighted in one of the Euro-MCD field studies [48]. Another important decision was to split the Euro-MCD 2.0 moral action domain and move it into the constructs of ethical competence and ethical climate, since these conceptually encompass actions [25, 49, 50]. Moreover, the term *ethical competence* was chosen because the concepts *ethical* and *moral* are treated as synonymous, and because ethical competence is more commonly used in the healthcare literature, as indicated by a PubMed search conducted in 2024.

**Table 2:**
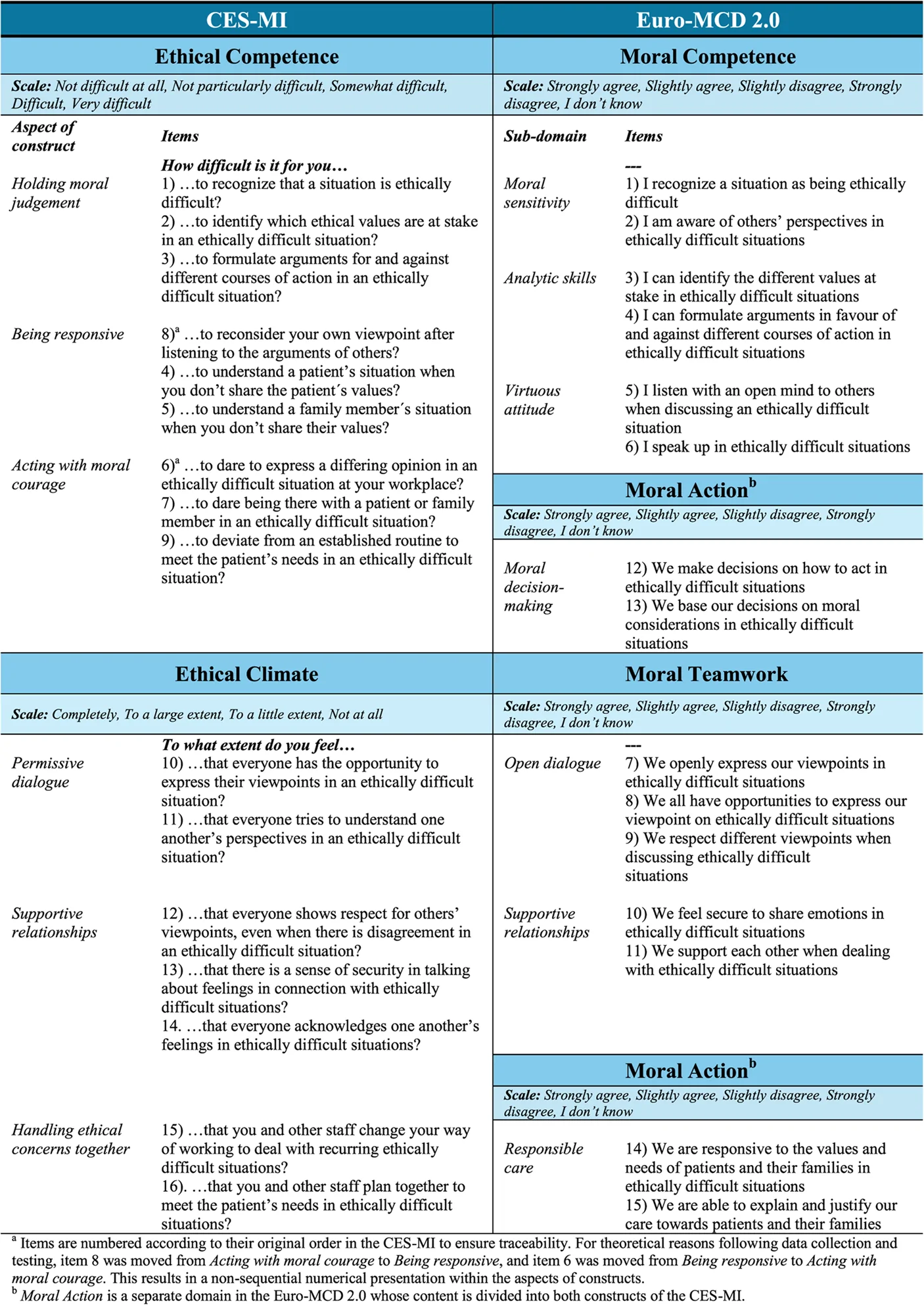
Content comparison between the CES-MI and the Euro-MCD 2.0

#### The ethical competence construct

The ethical competence construct encompasses the aspects holding moral judgement, being responsive, and acting with moral courage. This framing aligns with Eriksson et al.’s [30] tripartite model of knowing, being, and doing, which also underpins the moral competence domain in the Euro-MCD 2.0 [17]. To deepen this conceptualisation, Gallagher’s framework [33] was incorporated, emphasising ethical perception (to see), ethical character (to be), and ethical action (to bring out ethical practice). These aspects are further supported by literature addressing ethical awareness, value recognition, moral reasoning, character strength, and the capacity to act ethically [49, 51, 52].

Given the inherent complexity of ethical challenges, the measurement scale was designed to facilitate honest self-reflection, particularly regarding the perceived difficulty of identifying ethically difficult situations. To encourage openness, even among respondents who may hesitate to acknowledge uncertainty, the response scale and associated items were formulated to normalise difficulty. Accordingly, respondents are invited to assess how challenging something is, rather than interpret difficulty as a personal weakness. A five-point unipolar scale ranging from *Not difficult at all* to *Very difficult* was therefore adopted.

##### Aspects and items of the ethical competence construct

*Holding moral judgement* builds on knowing [30] and to see [33], reflecting Lechasseur’ et al.’s [49] emphasis on philosophical, theoretical, and practical knowledge while acknowledging intrinsic ambiguities. A key focus was practical wisdom, described by Aristotle as the virtue of deliberating well (phronesis). This underscored that knowledge alone is insufficient, prompting refinement of the Euro-MCD 2.0 subdomains analytical skills and moral sensitivity. From this, holding moral judgement emerged as a potential aspect, influenced by Bornemark’s [53] account of moral judgement with its emphasis on practical wisdom as outlined by Aristotle [51]. Bornemark [53] defines judgement as a skill marked by openness to not knowing, involving emotions, experiences, and theoretical insights about what matters. Broader descriptions include critical reflection on values, principles, needs, and beliefs, alongside open-mindedness, consistency, logic, clarity, and active listening [51]. The aspect holding moral judgement is operationalised in items 1–3 of the CES-MI, which address identifying ethically difficult situations, identifying values at stake, and formulating arguments for and against various courses of action.

*Being responsive* builds on Eriksson’s notion of being and Gallagher’s concept of to be. In the literature, related ideas are expressed through terms such as moral character [54], character strength [51], ethical behaviour [49], moral attitude [16], and virtuous attitude [17]. Moral character encompasses traits such as altruism, honesty, and forgiveness [54], while character strength includes temperance, kindness, humanity, empathy, compassion, and bravery [51]. Ethical behaviour has similarly been described in terms of moderation, respect, responsiveness, and attentiveness to vulnerability [49]. The literature suggests that virtues and moral actions are closely intertwined, suggesting that ethical behaviour and character strength inherently require balancing the aspiration to be a good person with the capacity to act rightly [49, 51]. Thus, virtues and actions appear interdependent in the development of ethical competence. Although the original Euro-MCD subdomain moral attitude [16] was considered, courage and responsiveness emerged as the virtues most central to ethical reflection and patient care. Notably, courage has been identified as particularly connected to moral action [54]. Items 4 (understanding a patient’s situation) and 5 (understanding a family member’s situation) were formulated to reflect responsiveness in caring. To reduce socially desirable responses, the phrase “when you don’t share the patient’s values” was added. These items generated substantial discussion, particularly concerning the wording “understanding their situation”, which underwent several revisions ranging from “being responsive to values and needs” to “imagining the patient’s or family member’s situation”. Item 8, which concerns reconsidering one’s viewpoint after listening to the others’ arguments, incorporates the active-listening aspect from item 5 in Euro-MCD 2.0, which emphasises listening with an open mind. The revised formulation strengthens the articulation of one’s own position while also reducing susceptibility to socially desirable responding.

*Acting with moral courage* draws on Eriksson’s notion of doing [30] and Gallagher’s concept of bringing out ethical practice [33]. However, Eriksson’s emphasis on rules and principles was not adopted; instead, the interdependence between virtues and moral actions was further explored. Responsibility was initially identified as the central virtue, consistent with the Euro-MCD 2.0 subdomain responsible care. Moral responsibility has been described as the desire to act rightly and to take responsibility by fulfilling duties, being accountable, and assuming ownership even in situations of moral uncertainty [51, 55]. The literature also conceptualises moral integrity as upholding moral convictions despite potential negative consequences [55, 56], a quality closely linked to courage, which is considered an essential component of moral decision-making [54]. Notably, in a qualitative CES study, courage was perceived by healthcare professionals as particularly important [57]. Item 6, addressing the willingness to express a differing opinion at work, and item 7, concerning daring to be present with a patient or family member, both reflect aspects of courage. Derived from Euro-MCD 2.0 item 6 (daring to speak up), these items were reformulated into more demanding actions to better capture concrete manifestations of moral courage.

Item 9, which focuses on deviating from an established routine to meet a patient’s needs, evolved through several iterations. It ultimately came to capture the idea of standing up for a patient, even when doing so involves challenging superiors, rules, or guidelines, or advocating for what one believes to be in the patient’s best interest. Such deviating from routine was therefore interpreted as an expression of both courage and responsiveness.

#### The ethical climate construct

The ethical climate construct was conceptualised as the perceived organisational environment that enables or constrains ethical behaviour and decision-making [25, 31, 50, 58]. Building on foundational work [31, 32, 59], ethical climate was understood as an aggregate of individual perceptions, typically represented through mean scores at the group or organisational level. The construct comprises three aspects: *permissive dialogue*,* supportive relationships*, and *handling ethical concerns together.*

*Permissive dialogue* was conceptualised using Habermas’ theory of communicative action, which underscores inclusive, non-coercive communication aimed at mutual understanding [34]. *Supportive relationships* were informed by Benhabib’s communicative ethics and her emphasis on mutual recognition, vulnerability, and interdependence as moral foundations [35]. The third aspect, *handling ethical concerns together*, reflects collaborative engagement in ethical reflection and decision-making at the team level, as emphasised by Lechasseur et al. [49].

To ensure that respondents answered items in the ethical climate construct from their individual perspectives, rather than on behalf of the entire group, an introductory phrase was added before the items: “To what extent do you feel…”. During the development phase, both the items and the response format were refined. All items were positively framed to capture the presence of ethical conduct. A four-point unipolar response scale, ranging from *Completely* to *Not at all*, was chosen because respondents found it difficult to judge whether the ethical climate was ‘sufficient’, and because many aspects of ethical climate are not directly observable. The scale enabled graded assessments, avoided a neutral midpoint to reduce ambiguity, and encouraged more decisive responses regarding the extent to which ethical norms were perceived as embedded within the organisation.

##### Aspects and items of the ethical climate construct

*Permissive dialogue* was selected as a central concept because it aligns with democratic dialogue, in which participants feel supported in raising and discussing ethical challenges [60]. It is described as a safe, respectful, and open communication climate where healthcare professionals can express diverse perspectives, emotions, and ethical concerns without fear of judgment or hierarchical pressure [61]. Permissive dialogue is considered a vital element of effective CES and a foundation for team cohesion and ethical reflection, particularly in interprofessional healthcare teams [57, 62]. Item 10, concerning opportunity to express one’s viewpoints, was developed from items 7–8 in Euro-MCD 2.0 and rephrased to fit the CES-MI item format (from statement to question) without altering the original meaning. Item 11, addressing the effort to understand one another’s perspectives in ethically difficult situations, was adapted from item 9 in Euro-MCD 2.0, which focused on mutual support in such situations. In the CES-MI, the focus was shifted towards understanding others’ perspectives without requiring agreement with their opinions, preferences or values, thereby more accurately reflecting the underlying concept of permissive dialogue.

*Supportive relationships* are manifested through communicative deliberations in which individuals remain aware of one another’s viewpoints [35]. Items within this aspect highlight that caring, sharing emotions, and respecting diverse perspectives are essential components for supportive relationships. Previous research has demonstrated a positive association between caring environments and psychological well-being [59]. In one study, participants reported becoming more emotionally connected and caring more closely for another within the team following MCD interventions [48]. Item 12, which concerns showing respect for others’ viewpoints even when there is disagreement in ethically difficult situations, was developed from item 9 in Euro-MCD 2.0. The CES-MI expands the focus to include mutual respect for differing viewpoints despite disagreement. Item 13, addressing the sense of security in talking about feelings related to ethically difficult situations, was changed linguistically adapted from item 10 in Euro-MCD 2.0 to fit the CES-MI item format (from statement to question) without altering the underlying meaning. Item 14, concerning acknowledging one another’s feelings in ethically difficult situations, was developed from item 11 in Euro-MCD 2.0. This adaptation reflects the understanding that permissive dialogue enables deeper forms of emotional support through shared feelings.

*Handling ethical concerns together* regards the ability of professionals to communicate and reach common understandings of how to act and how to approach each other in ethically challenging situations. According to Benhabib [35], communicative ethics calls for identifying a variety of action alternatives rather than limiting deliberation to what each individual deems acceptable. Within such a communication community, participants can reverse perspectives, identify different viewpoints, and balance competing moral standpoints. These communicative processes support the development of sound moral judgments regarding possible courses of action [35]. In this aspect of the construct, the focus was on capturing not only how communication promotes joint deliberation on appropriate actions and decisions for patients and families, but also how shared understandings of the patient’s situation are formed. This informed the development of an item addressing common understandings of how to handle ethical concerns collectively. Observation studies support this rationale: Bartholdson et al. [10] found that care practices became more coherent as perspectives were clarified during CES, while Grönlund et al. [63] observed that professionals shifted from individual to shared understandings through interprofessional communication about ethical challenges. Item 15, which concerns how professionals adapt their way of working to manage recurring ethically difficult situations, was derived from items 12 and 13 in Euro-MCD 2.0. The item was developed to address not only decision-making based on moral considerations, but also whether professionals collectively reconsider and adjust their actions over time. Item 16, addressing whether professionals plan together to meet the patient’s needs in ethically difficult situations, was derived from items 14 and 15 in Euro-MCD 2.0. In this item, particular emphasis was placed on the patient’s needs, recognising that these do not always coincide with the needs of families.

### Phase 2 – cognitive evaluation

#### Cognitive interviews

Cognitive interviews revealed challenges in identifying the precise position on the response scale in relation to specific clinical situation. The scale format itself was consistently described as clear, intuitively structured, and easy to comprehend. Participants reported difficulty interpreting the intended meaning of *others* in the item “to empathize with others’ situation when you don’t share their values” The term could be understood as referring to patients, healthcare professionals, family members, or even bystanders: “Yes, in this case, … the other is the person seeking help. It can also be, what do you call it, the relatives around them. It could be that too” (participant 1). To address this ambiguity, the item was clarified and divided into two separate items (items 4 and 5 in the CES-MI), distinguishing between understanding the patient’s situation and understanding the family members’ situation. Item 7 in the CES-MI, focusing on daring to be present with a patient or family member in an ethically difficult situation, was initially formulated as two separate items: “To dare to be present in the patient’s difficult situation” and “To dare to be present in the family’s difficult situation.” However, the cognitive interviews revealed several sources of uncertainty. Participants tended to include the family when responding to the patient-focused item, despite the intended distinction. Additionally, because the items did not explicitly reference ethically difficult situations, one participant interpreted the question as a general assessment of empathetic ability: “Within healthcare, an empathetic ability is valuable to have. However, trying to understand how it can be seen from their perspective, and then perhaps - yes, then perhaps in some way you can change and share” (participant 4). Another participant, who more readily referred to ethically difficult situations, found the item easier to interpret. Three of the four participants also struggled to determine which family the item referred to: “To dare to be present in the family’s difficult situation. So, is it my own family… Or is it the patient’s family?” (participant 3). Based on these findings, the two items were merged and clarified into a single item addressing “to dare being there with a patient or family member in an ethically difficult situation.”

#### Written open-ended inquiry

The open-ended written inquiry yielded descriptions of 28 unique ethically challenging situations. Many responses involved complex decision-making, particularly conflicts concerning autonomy and differing opinions between patients, relatives or other healthcare professionals about the best course of action, especially when the patient’s own voice risked being marginalised. As one participant described: “When I have an opinion about what is best for the patient, relatives or other healthcare professionals around the patient may have an opposite opinion about what they think is best for the patient. The worst is if the patient is not involved in the decisions.” Additional responses described challenges related to communication across cultural boundaries; tensions between professional integrity and personal values; and situations in which clinical guidelines did not align with the perceived needs of the patient.

### Phase 3 – psychometric evaluation

Responses to the CES-MI were used to evaluate the instrument’s psychometric properties. The final version of the CES-MI comprises 16 items: nine in the ethical competence construct and seven in the ethical climate construct. The proportion of missing data or incomplete responses was low (4%). Item and person locations indicated appropriate targeting, with no evidence of misfit or disordered thresholds. Reliability indices, including the Person Separation Index, Ordinal alpha and Cronbach’s alpha, were consistently high, supporting strong internal consistency and the instrument’s ability to distinguish between levels of the latent traits. Unidimensionality was confirmed through parallel analysis, and no differential item functioning (DIF) was detected across sex or intervention groups, demonstrating measurement invariance. Table 3 present measurements properties of the CES-MI constructs, followed by a detailed description of selected analyses.

**Table 3:** Measurements properties of the CES-MI constructs

Measurement properties	Constructs	
	Ethical competence	Ethical climate
**Items**		
Location, mean (SD)	0.00	0.00
Fit residual, mean (SD)	-0.04 (1.28)	-0.62 (1.11)
**Persons**		
Location, mean (SD)	-1.02 (1.14)	-1.28 (1.90)
Fit residual, mean (SD)	-0.33 (1.12)	-0.48 (0.98)
**Total item trait interaction**		
Total item *x*^2^ (df) *p-value*	27.92 (18) *0.06*	21.92 (14) *0.08*
**Reliability**		
Person separation index	0.88	0.81
Ordinal alpha	0.83	0.89
Cronbach’s alpha (SD)	0.79 (0.49)	0.81 (0.4)
**Item fit**	*Item range*: − 0.887–1.772	*Item range*: -1.706–2.880
**Local dependency**	3 local dependencies	3 local dependencies
**Unidimensionality**	*Parallel analysis* = 1 dimension. *Eigenvalues* (4.1, 1.1 & 1.1). *Scree plot* 1 component	*Parallel analysis* = 1 dimension. *Eigenvalues* (4.16 & 1.16). *Scree plot* 1–2 components
**Response category functioning**	No disordered item thresholds	No disordered item thresholds
**Reliability**	0.79	0.81
**Differential item functioning (DIF)**	0 DIF (Sex and intervention)	0 DIF (Sex and intervention)

*SD* Standard deviation, *df *Degrees of freedom

**Fig. 2:**
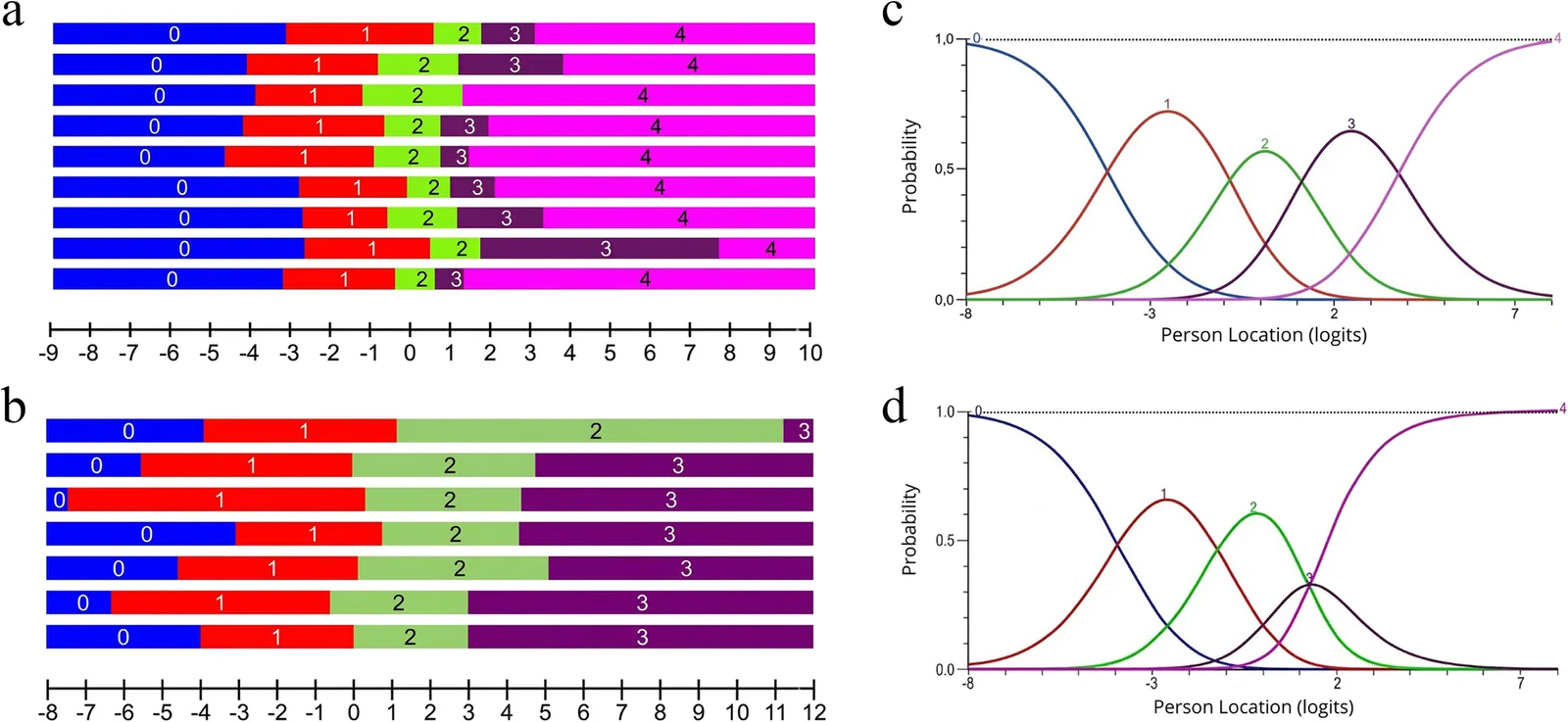
**a** Thresholds for all items in the ethical competence construct **b** Thresholds for all items in the ethical climate construct **c** Thresholds for item 2 in the ethical competence construct. Each coloured category probability curve represents the probability (y-axis) of responding in that response category relative to various estimated person logit locations (x-axis) **d** Thresholds for item 3 in the ethical competence construct. Each coloured category probability curve represents the probability (y-axis) of responding in that response category relative to various estimated person logit locations (x-axis)

According to the residual fit statistics, all items demonstrated good fit to the Rasch model (Table 3). A graphical inspection of the item characteristic curves confirmed good agreement between the observed data and the Rasch model for all items. Thresholds for all items are presented in Fig. 2a and b, whereas Fig. 2c provides a representative example of the category probability curves for the majority of items. One threshold in item 3 in the ethical competence construct (Fig. 2d) displayed a slightly flatter curve compared with the other items, but the deviation was minor. The mean item residual correlations were − 0.121 for ethical competence and − 0.161 for ethical climate. Based on these values, the critical thresholds for identifying local dependency were 0.099 for the ethical competence scale and 0.059 for the ethical climate scale. Using these thresholds, local dependency was detected between the following item pairs in the ethical competence construct: items 1 and 2 (0.381), items 2 and 3 (0.119), and items 4 and 5 (0.555). For the ethical climate construct, local dependency was identified between items 2 and 3 (0.144), items 4 and 5 (0.07), and items 6 and 7 (0.278).

**Fig. 3:**
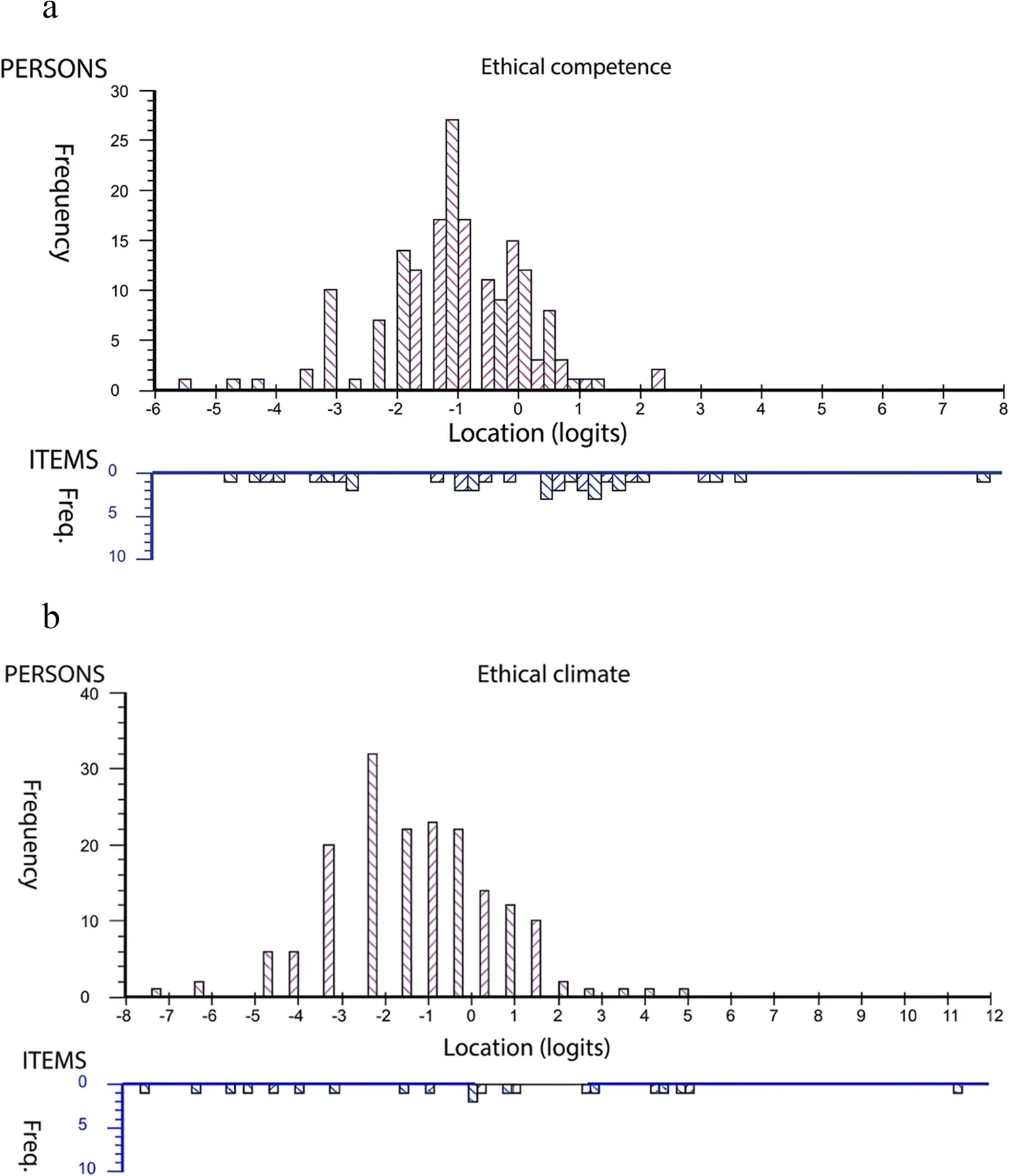
**a** The person-item threshold distribution for the ethical competence construct **b** The person-item threshold distribution for the ethical climate construct

The analysis identified two outlying category thresholds. Threshold 4 for item 8 (Fig. 3a), which assesses ethical competence, showed an outlying value, indicating that the corresponding response category was rarely used or may not have functioned as intended. Similarly, threshold 3 in item 1 (Fig. 3b), within the ethical climate construct, displayed an outlying value. These findings suggest potential issues with the functioning of specific response categories, possibly due to ambiguity or limited relevance for respondents.

Figure 3a The person-item threshold distribution for the ethical competence construct.

## Discussion

Based on the findings, the CES-MI appears to be a trustworthy measurement of perceived ethical competence and ethical climate in the context of CES in healthcare. The evaluated aspects converged to form coherent, well-defined constructs with strong psychometric properties indicating that ethical competence and ethical climate can be assessed independently. It is likely that an ethical climate promotes ethical competence of the individuals. However, individuals might have ethical competence despite a poor ethical climate even though it could hamper the possibility to use one’s ethical competence. Both constructs demonstrated clear structure, high reliability, and good sensitivity to differences between respondents, indicating that CES-MI can distinguish varying levels of ethical competence and ethical climate in practice. Importantly, the instrument demonstrated stable functioning within the study population, supporting its potential as a promising instrument for use in pre-hospital care settings. Below, we discuss the overarching conceptual framework, user-friendliness, as well as psychometric properties of the CES-MI. We thereafter bring these components together into an integrated and coherent account for each construct.

### A conceptually grounded instrument?

Conceptually, we argue that the constructs of ethical competence and ethical climate are coherently delineated through the specific aspects that constitute each construct, albeit within the context of CES. In contrast to the Euro-MCD 2.0 instrument, moral action is not conceptualised as a separate aspect, instead it appears as action-oriented aspects embedded within each construct in CES-MI. This approach aligns with the work of Lechasseur [49], Kulju [51] and Cullen [32]. Nevertheless, additional outcomes may be important when evaluating CES. One potential outcome concerns the management of moral distress. However, prior studies have yielded mixed findings; some report that CES can reduce moral distress in ethical decision-making [64], whereas others have not observed reductions in moral distress following a series of CES sessions [65, 66]. Furthermore, moral distress could be seen as a sign of moral sensitivity and thus, a reduction or elimination might not be entirely desirable [19, 67]. Instead, resilience has been proposed as a more appropriate indicator of how individuals and organisations cope with moral distress [65, 68]. CES may help foster an environment conducive to the development of moral resilience [65, 66, 69].

### A well-understood and usable instrument?

Previous research has questioned the reliability of scales that risk social desirability response bias, particularly their tendency to conflate transient response styles with stable personality traits [26, 28], as well as the use of evaluative item wording that may elicit self-protective responses rather than accounts of lived experience [70, 71]. Moreover, social desirability represents a particular methodological concern in morally charged contexts [8, 9, 26], encompassing both impression management, deliberate attempts to present oneself favourably, and self‑deception, reflecting positively biased yet unconscious self‑perceptions [8, 27]. To mitigate these risks, evaluative connotations were minimised using neutral language, and evaluative pressure was reduced in the construction of the response scales [71, 72]. Attention was devoted to linguistic calibration, including pronoun choice, item framing, and construct-specific introductory phrasing [5, 8, 9, 28].

Cognitive interviews did not reveal difficulties in understanding the response scale; reported challenges instead concerned identifying the precise position on the response scale in relation to specific clinical situation. Accordingly, unipolar response scales with a reasonable number of options and an appropriate level of precision were adopted, balancing natural responding with sufficient response variability in line with established measurement principles [70, 73, 74]. Respondents also noted that the heterogeneity of clinical situations occasionally complicated the determination of an exact response option. Nevertheless, the scale format itself was consistently described as clear, intuitively structured, and easy to comprehend. This pattern suggests that any response difficulties stemmed primarily from the contextual complexity of everyday clinical work rather from the scale design.

### A psychometric reliable and consistent instrument?

The psychometric evaluation indicate that all proposed items demonstrated good Rasch model fit, functioning as intended by forming interpretable unidimensional scales within each construct and meeting recommended psychometric criteria. The instrument appears to address challenges related to evaluative pressure [27, 28] and to support the generation of authentic responses. Although some local dependencies between items were observed in the psychometric evaluation, they were limited in scope and did not compromise the overall measurement properties. Taken together, the findings indicate that both scales meet key psychometric standards and provide a robust foundation for subsequent analyses.

### A coherent construct of ethical competence?

The construct of ethical competence originated from Eriksson’s framework of knowing, being and acting [30] as well as Gallagher’s framework [33] and several other’s conceptual work [49, 51, 52]. Another similar framework is the four components of acting morally by Narvaéz and Rest [75], which describes four steps that determine and constitute the basis for moral actions. Furthermore, it proved impossible to retain mutually exclusive aspects of the construct of ethical competence. One reason is that the construct incorporates latent aspects that reflect virtues spanning all three areas: (1) holding moral judgement as an Aristotelian virtue linked to knowledge, (2) being responsive as a relational-ethical virtue, and (3) acting with moral courage as a virtue associated to action. Despite this interdependence, the conceptualisation of moral judgement appears robust and adds depth to the Euro‑MCD 2.0 subdomains of moral sensitivity and analytical skills. Greater challenges emerged in relation to the aspect of being responsive. Relocating the virtue of courage to the action‑related aspect of construct also resulted in a narrower interpretation than in the Euro‑MCD 2.0 subdomain of virtuous attitude and the original instrument’s moral attitude.

Because aspects of ethical competence are at a latent level, they carry an elevated risk of eliciting socially desirable responses. To reduce tendencies toward self‑image protection [70, 71] and to normalise the inherent complexity of ethical challenges, the introduction to this construct deliberately employed the term *difficulty*. This linguistic choice is intended to foster more candid and contextually grounded responses. The aspects concerning the capacity to hold moral judgement and to act with moral courage raise additional concerns related to self‑deception. Identification of an ethically difficult situation may be overestimated when ethical knowledge is limited and may, paradoxically, increase following CES participation, as heightened awareness of ethical complexity can result in perceiving a greater number of situations as ethically challenging.

In the ethical competence construct, a local dependency was identified between item 4 and item 5, indicating partial content overlap. These items had previously been revised following cognitive interviews, where it became evident that respondents struggled to define who ‘others’ referred to. To address this ambiguity, the original item ‘to empathize with others’ situation when you don’t share their values’ was split into two distinct items (item 4 and 5) - one referring to ‘patient’ and the other to ‘family’. The dependency was resolved when the items were combined, supporting the conclusion that the revised formulation provides a clearer and more accurate representation of the intended construct. The final formulation thus balances conceptual clarity with psychometric validity. Although some local dependencies were observed, they were limited and did not compromise the overall measurement properties.

### A coherent construct of ethical climate?

The construct of ethical climate in the CES-MI was derived and redefined from the moral teamwork domain of the Euro-MCD 2.0. In Euro-MCD 2.0, moral teamwork was presented as a pragmatic concept describing how professionals work and interact when encountering ethical challenges [17]. In the development process of CES-MI, ethical climate was considered a more suitable concept, grounded on the one hand in theoretical work by Cullen [59] and Olson [31, 58], and on the other hand understood as a comprehensive construct linked to shared organisational values, leadership practices, and the ethical approach adopted by professionals.

The Euro-MCD 2.0 subdomain open dialogue was replaced by permissive dialogue in the CES-MI. Whereas open dialogue originally emphasised open and honest dialogue conducted in an equal and respectful manner [17], permissive dialogue was found to capture a broader set of conditions necessary for achieving shared understandings. These conditions include respect for differing viewpoints as well as the creation of space that enables genuine responsiveness among group members. Previous studies have shown that participants arrive at shared understandings of ethical challenges through permissive dialogue in CES settings [62, 76]. The aspect supportive relationships were retained from Euro MCD 2.0 [17], although the item content was expanded to encompass not only safety and mutual support but also respect for differing perspectives in situations of disagreements and acknowledgment of emotions in ethically difficult circumstances. This broader orientation represents a more comprehensive view of a caring and supportive environment.

In Euro-MCD 2.0, the former domain moral action was divided into two separate aspects of construct in the CES-MI to distinguish more clearly between individual and collective perspectives. Accordingly, the CES-MI places acting with moral courage within the construct of ethical competence, while handling ethical concerns together is situated within the construct of ethical climate. In Euro-MCD 2.0, moral action was conceptualised as good interactions, responsiveness to patients and their families, and the ability to justify care-related decisions [17]. The process of handling ethical concerns together concerns, on the one hand, the capacity to value and revalue one’s own standpoints to arrive at shared understandings about how ethical challenges should be approached and how patients’ needs can best be met. On the other hand, it involves collective deliberation aimed at identifying appropriate courses of action for addressing ethical challenges. Silén et al. [24] describes that participants perceived that regular CES enhanced the ethical climate, and Grönlund et al. [77] reported that participants were supported to reach a democratic dialogue through CES-based reflection.

Regarding the measurement and response scale in the CES-MI ethical climate construct, grading extent was selected for its neutrality, emphasising subjective experience rather than normative judgement. This choice is consistent with the original Euro-MCD instrument [16]. In Euro-MCD 2.0, items within the moral teamwork domain were framed as *we* statements [17], which may be interpreted as referring to collective team functioning. However, healthcare professionals frequently hold divergent experiences within the same organisational environment, making it difficult to respond on behalf of the group. Koskenvouri [78] similarly notes that individuals may perceive the ethical climate differently and experience varying effects of organisational conditions on morale and attitudes. Prior research further indicates that direct questions concerning personal attitudes can heighten socially desirable responding [8, 9, 28]. For these reasons, CES-MI employs *you* statements to capture respondents’ personal experiences while reducing pressure to align responses with perceived team norms or idealised expectations.

Ethical climate is conceptualised as a construct operating simultaneously at organisational and individual levels, a duality that contextualises the psychometric results [78–80]. At the organisational level, ethical climate encompasses shared norms, expectations, and collective perceptions that shape decision-making and ethical behaviour. At the individual level, it is interpreted through personal values, moral identity, and demographic characteristics, thereby influencing attitudes, well-being, and responses to ethical challenges [79, 81]. Multilevel studies emphasise that ethical climate emerges from aggregated individual perceptions while also being shaped by structural and cultural characteristics of the organisation [80, 82]. This dual character is particularly relevant in healthcare environments, where individual experiences and organisational ethics continually interact [78, 79, 81].

Interpreted within this theoretical frame, the factor-analytic results provide meaningful insight. Items 15 and 16 loaded similarly on both factors after rotation. Rather than indicating a measurement weakness, this pattern reflects the inherent multilevel nature of ethical climate; because it is shaped by both group-level conditions (e.g., workplace norms and culture) and individual interpretation, certain items naturally capture elements of both dimensions. The cross-loadings therefore illustrate the close interrelation between individual and collective aspects; distinct yet strongly connected components of how ethical environments are perceived and enacted. This interpretation aligns with previous literature describing ethical climate as neither exclusively individual nor organisational but instead a dynamic interaction between these levels [78–82].

### Strengths, limitations and future directions

A strength of the study is the extensive conceptual groundwork and the rigorous revision process. This underscores the complexity of developing an instrument that measures latent constructs. It could be argued that ethical competence and ethical climate are domains of an overarching construct. However, in CES-MI they are treated as distinct constructs because they operate at the individual and group levels. Treating them as domains (subscales) and merging the scores into a total CES outcome score would be reductive and risk overlooking other relevant outcomes, such as moral distress. One limitation might be that aspects of constructs were formalised relatively late, as earlier stages primarily concentrated on drafting items. However, this approach does not appear to have compromised the instrument’s robustness, as the subsequent process involved extensive, iterative, and theory-informed dialogue. This rigorous refinement process strengthens the coherence and supports the trustworthiness of the instrument. Although the instrument performed well both psychometrically and conceptually, further refinement is warranted, particularly for items that may be susceptible to self-deception or social desirability bias. A limitation regarding the cognitive interviews is the small sample size of four participants. Furthermore, these individuals were highly experienced, which may have influenced their interpretation of the items. While older or more experienced professionals might reflect a higher level of ethical competence, it is also possible for individuals to possess high ethical competence despite a younger age or limited clinical experience. This potential discrepancy underscores the need for further testing with a larger and more diverse sample to ensure construct validity across all levels of clinical experience. Additionally, items 4 and 5 did not undergo cognitive testing. For these items, additional attention could be directed toward the ‘being responsive’ aspects, particularly the challenge of understanding a family member’s (item 4) or a patient’s (item 5) situation when their values differ from those of the respondent. For item 5, replacing situation with perspective may better reflect the interpretative stance respondents take toward family members. Item 4 may require more substantial revision, such as replacing understand with be attentive to and reframing situation as the patient’s needs or what matters to the patient. Such modifications may strengthen the instrument’s alignment with relational ethics [83]. Moreover, further qualitative work is needed, for example by additional cognitive interviews in more diverse groups to deepen the understanding of how different respondents interpret and answer each item in the CES‑MI.

DIF was evaluated only for sex and the intervention group. Ideally, DIF should also be examined for additional variables such as age and work experience. Selection bias cannot be ruled out, as the study design did not allow for dropout analysis. Nevertheless, the sample demonstrated variation in age, sex, education, and work experience, and met recommended sample-size guidelines for polytomous Rasch models (10–20 participants per threshold) [41].

Although the study had a minor missing data rate of 4%, this was not considered to pose a significant risk to the findings. Missing data can affect item parameter estimation and model fit in Rasch analysis, particularly when missingness is not completely random [84–86]. However, given that the total sample of 176 respondents still substantially exceeded the requirements for all CES-MI dimensions, which correspond to a minimum of 120 for holding moral judgment, being responsive, and acting with moral courage; 90 for supportive relationships; and 60 for permissive dialogue and handling ethical concerns together. Consequently, the impact on the stability of the estimates is negligible. Furthermore, to avoid potential bias from case deletion [86], all valid responses were retained in accordance with standard procedures in RUMM2030, which effectively handles missing values without compromising the instrument´s robustness. Moreover, while the current study used principal component analysis for initial exploration of the instrument’s structure, future research should employ confirmatory factor analysis to rigorously test and verify the stability of the factor structure across diverse populations.

Finally, a limitation is that all authors involved, except one, were registered nurses. However, the nurses were ethics researchers with diverse practice backgrounds across multiple healthcare settings, which likely strengthens the instrument’s ecological validity and transferability, particularly given the central role nurses play in CES [48].

Moreover, the respondents represented a diverse range of professional groups but were all from a single clinical setting. The ambulance clinicians worked in a pre-hospital setting in small teams which could have influenced how the ethical climate was perceived. However, also in pre-hospital settings clinicians belong to a larger team at a specific unit where the ethical climate is important to deal with ethical challenges. To strengthen transferability, especially regarding ethical climate, further studies evaluating the instrument need to be conducted in more contexts and countries.

## Conclusions

The CES-MI is a coherent, theoretically grounded, and psychometrically robust instrument for measurement in the CES context, suitable for both clinical practice and research. The findings indicate that ethical competence and ethical climate can be assessed independently, depending on evaluative needs. The strong validity likely reflects the extensive qualitative and psychometric work underpinning the CES-MI, together with a user-friendly response format that supports authentic and reliable self-assessment. A next step is to examine the instrument’s sensitivity to change over time as well as to evaluate its applicability across diverse healthcare settings. Additionally, incorporating open-ended questions for selected items may enhance insight into concrete changes, while asking respondents to rate their experiences of change also may strengthen the assessment of change over time.

## Supplementary Information

Supplementary Material 1.

## Data Availability

Data from the instrument testing and psychometric evaluation are available from the corresponding author on reasonable request.

## Abbreviations

CES: Clinical Ethics Support
CES-MI: Clinical Ethics Support Measurement Instrument
DF: Degrees of Freedom
DIF: Differential Item Functioning
EMT: Emergency Medical Technician
Euro‑MCD: European Moral Case Deliberation Outcomes Instrument
MCD: Moral Case Deliberation
RN: Registered Nurse
SD: Standard Deviation
