# Supplementary material for: Assessing ethical competence and ethical climate: development and psychometric testing of the Clinical Ethics Support Measurement Instrument (CES-MI)
Source: BMC Med Ethics. 2026 Sep 8;27:207. doi: 10.1186/s12910-026-01573-6 (PMC13621694; doi:10.1186/s12910-026-01573-6)
Supplement: Supplementary file 1 [file 12910_2026_1573_MOESM1_ESM.pdf]

Supplementary file 1: CES-MI

Ethically difficult situations in healthcare

Think about situations that you have experienced as ethically difficult, that is, when you have experienced uncertainty about what is right/wrong or good/bad.

Part A: Questions about how it is for you

How difficult is it for you…	Not difficult at all	Not particularly difficult	Somewhat difficult	Difficult	Very difficult
1. … to identify that a situation is ethically difficult?	☐	☐	☐	☐	☐
2. ... to identify which ethical values are at stake in an ethically difficult situation?	☐	☐	☐	☐	☐
3. ... to formulate arguments for and against different courses of action in an ethically difficult situation?	☐	☐	☐	☐	☐
4. … to understand a patient's situation when you don't share the patient´s values?	☐	☐	☐	☐	☐
5. … to understand a family member´s situation when you don't share their values?	☐	☐	☐	☐	☐
6. … to dare to express a differing opinion in an ethically difficult situation at your workplace?	☐	☐	☐	☐	☐
7. … to dare being there with a patient or family member in an ethically difficult situation?	☐	☐	☐	☐	☐
8. … to reconsider your own viewpoint after listening to the arguments of others?	☐	☐	☐	☐	☐
9. … to deviate from an established routine to meet the patient's needs in an ethically difficult situation?	☐	☐	☐	☐	☐

If you have anything you would like to add, please do so below:

____

____

____

____

Part B: Questions about how it is at your workplace

Think of all healthcare professionals at the workplace where you work the most.

**To what extent do you feel…**	Completely	To a large extent	To a little extent	Not at all
10. … that everyone has the opportunity to express their viewpoints in an ethically difficult situation?	☐	☐	☐	☐
11. … that everyone tries to understand one another's perspectives in an ethically difficult situation?	☐	☐	☐	☐
12. … that everyone shows respect for others' viewpoints, even when there is disagreement in an ethically difficult situation?	☐	☐	☐	☐
13. … that there is a sense of security in talking about feelings in connection with ethically difficult situations?	☐	☐	☐	☐
14. … that everyone acknowledges one another's feelings in ethically difficult situations?	☐	☐	☐	☐
15. ... that you and other staff change your way of working to deal with recurring ethically difficult situations?	☐	☐	☐	☐
16. … that you and other staff plan together to meet the patient's needs in ethically difficult situations?	☐	☐	☐	☐

If you have anything you'd like to add, please do so below:

____

____

____

____
